# Investigation of *Diospyros* Kaki L.f husk extracts as corrosion inhibitors and bactericide in oil field

**DOI:** 10.1186/1752-153X-7-109

**Published:** 2013-07-01

**Authors:** Jie Zhang, Yingpan Song, Huijun Su, Li Zhang, Gang Chen, Jingrui Zhao

**Affiliations:** 1 College of Chemistry and Chemical Engineering, Xi’an Shiyou University, Xi’an Shaanxi 710065, People’s Republic of China; 2 Shannxi Hai’an Industry Co., LTD, Xi’an Shaanxi 710065, People’s Republic of China

**Keywords:** Persimmon, Acid corrosion, Weight loss, Microbiological corrosion

## Abstract

**Background:**

Hydrochloric acid is used in oil-well acidizing commonly for improving the crude oil production of the low-permeable reservoirs, while it is a great challenge for the metal instruments involved in the acidification. Developing natural products as oilfield chemicals is a straight way to find less expensive, green and eco-friendly materials. The great plant resources in Qin-ling and Ba-shan Mountain Area of Shannxi Province enable the investigating of new green oil field chemicals. *Diospyros* Kaki L.f (persimmon), a famous fruit tree is widely planted in Qin-ling and Ba-shan Mountain Area of Shaanxi Province. It has been found that the crude persimmon extracts are complex mixtures containing vitamins, *p*-coumaric acid, gallic acid, catechin, flavonoids, carotenoids and condensed tannin and so on, which indicates the extracts of persimmon husk suitable to be used as green and eco-friendly corrosion inhibitors.

**Findings:**

Extracts of persimmon husk were investigated, by using weight loss and potentiodynamic polarisation techniques, as green and eco-friendly corrosion inhibitors of Q235A steel in 1M HCl. The inhibition efficiency of the extracts varied with extract concentration from 10 to 1,000 mg/L. There are some synergistic effects between the extracts and KI, KSCN and HMTA. Potentiodynamic polarization studies indicate that extracts are mixed-type inhibitors. Besides, the extracts were screened for antibacterial activity against oil field microorganisms, and they showed good to moderate activity against SRB, IB and TGB.

**Conclusions:**

The inhibition efficiency of the extracts varied with extract concentration from 10 to 1,000 mg/L, and the highest reaches to 65.1% with the con concentration of 1,000 mg/L WE. KI, KSCN and HMTA they can enhance the IE of WE effectively to 97.3% at most, but not effective for KI and KSCN to AE. Tafel polarisation measurements indicate the extracts behave as mixed type inhibitor. Investigation of the antibacterial activity against oil field microorganism showed the extracts can inhibit SRB, IB and TGB with moderate to highly efficiency under 1,000 mg/L, which makes extracts potential to be used as bifunctional oil field chemicals.

## Introduction

**Figure 1:**
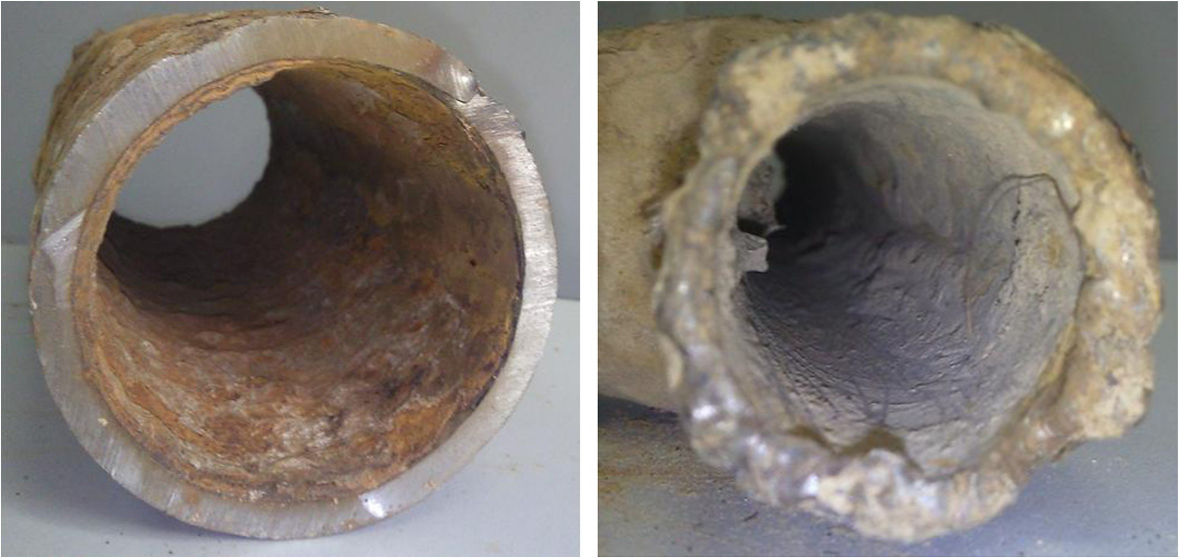
The corrosion in the re-injection pipes of the oil field.

Hydrochloric acid is used in oil-well acidizing commonly for improving the crude oil production of the low-permeable reservoirs [1], while it is a great challenge for the metal instruments involved in the acidification, as shown in Figure 1. Due to the aggressive nature of HCl and other corrosive factors, the practice of inhibiters is commonly used to reduce such corrosive attack [2]. Compounds, containing functional electronegative groups and abundant p-electron in conjugated double or triple bonds, are found to be very efficient as corrosion inhibitors [3]. It has been commonly recognized that an organic inhibitor usually promotes the formation of a chelate film on a metal surface by transferring electrons from the organic compounds to the metal and forming a coordinate covalent bond during the chemical adsorption, thereby resisting the corrosion in acidic solutions [4]. In present, the heteroatomic organic compounds as well as aromatic compounds are the widely used efficient corrosion inhibitors.

**Figure 2:**
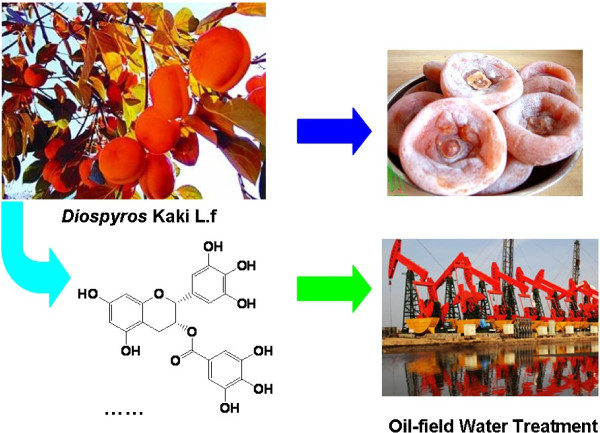
Development of persimmon extract as oil field chemical.

The great plant resources in Qin-ling and Ba-shan Mountain Area of Shannxi Province enable the investigating of new green oil field chemicals. *Diospyros* Kaki L.f (persimmon), a famous fruit tree is widely planted in Qin-ling and Ba-shan Mountain Area of Shaanxi Province. It has been found that the crude persimmon extracts are complex mixtures containing vitamins, *p*-coumaric acid, gallic acid, catechin, flavonoids, carotenoids and condensed tannin and so on [5], which indicates the extracts of persimmon husk suitable to be used as green and eco-friendly corrosion inhibitors. Moreover, microbiologically influenced corrosion (MIC) caused by oil field microorganisms, such as sulfate reducing bacteria (SRB), iron bacteria (IB), and total general bacteria (TGB), is also a considerable problem. As it has been reported that the compounds combine with proteins and show some antibacterial activities [6], the persimmon extracts are anticipated to be corrosion inhibitors and bactericides for oil field microorganisms in oil field water treatment, as shown in Figure 2.

The present work focuses on the assessment of persimmon extracts as corrosion inhibitors for Q235A steel in HCl solution. The corrosion protection effect and the mechanism of corrosion inhibition were investigated by means of linear DC polarization. The bioactivity against oil field microorganisms was also screened for the inhibition of MIC.

## Experimental

### Materials and methods

Persimmon husk, gathered in October 2011 in Qinling Mount, were washed by clean water and dried at 60°C. Then it was shattered into powders, and the powders were heated under reflux with water or alcohol for 4 h. The mixture was cool to the room temperature, and yellow solution was filtered, and solvent was removed to yield dry extract. The persimmon husk extracts obtained by use of water and alcohol were named as WE and AE respectively.

### Gravimetric measurements

The corrosion tests were performed on Q235A with a composition (in wt.%) C: 0.22, P: 0.045, Si: 0.35, S: 0.05, Mn: 1.40, and Fe balance. The electrolyte solution was 1 M HCl, prepared from analytical grade 38% HCl and distilled water. The concentrations of persimmon leaves extracts were employed as 10, 50, 100, 200, 500, and 1,000 mg/L. All tests have been performed in water solutions and at 60 ± 0.5°C for 5 h. The gravimetric tests were carried out according to the Standard of Petroleum and Natural Gas Industry of People’s Republic of China (Method of SY/T5273-2000, Evaluation method for behavior of corrosion inhibitor for produced water of oilfield) with a few modifications. Each test was done with three specimens simultaneously to give reproducible results.

### Electrochemical measurements

The electrodes were mechanically abraded with a series of emery papers (800 and 1,200 grades), then rinsed in acetone and double-distilled water before their immersion in the experimental solution. Electrochemical measurements were conducted in a conventional three-electrode thermostated cell. The electrode was inserted into a Teflon tube and isolated with polyester so that only its section (0.5 cm^2^) was allowed to contact the aggressive solutions. A platinum disk as counter electrode and standard calomel electrode (SCE) as the reference electrode have been used in the electrochemical studies.

The potentiodynamic curves were recorded using a CS350 system connected to a personal computer. The working electrode was first immersed in the test solution for 60 min to establish a steady state open circuit potential. After measuring the open circuit, potential dynamic polarization curves were obtained with a scan rate of 0.5 mV/s. Corrosion rates (corrosion current densities) were obtained from the polarization curves by linear extrapolation of the anodic and cathodic branches of the Tafel plots at points 100 mV more positive and more negative than the E_corr_.

CS350 electrochemical workstation hardware parameters:

Potentiostat potential control: ± 10 V; Current Control Range: ± 2.0A; Potential control precision: 0.1% × full scale reading ± 1 mV; Current control accuracy: 0.1% × full scale reading; Potential resolution: 10 μV (>100 Hz), 2 μV (< 10 Hz); Current resolution: < 10 pA; Potential rise time: < 1 μS (< 10 mA), < 10 μS (< 2A); Auxiliary 24-bit data acquisition-10 KHz, 20bit-1 KHz; Reference electrode input impedance: 1012 ohms || 20 pF; Current range 2A-00 nA, a total of 8 files; Tank pressure: 21 V; CV and LSV scan rate: 0.01-20000 mV/s; CA and CC pulse width: 0.0001-1000 s; Potential scan potential incremental: 0.1 mV-1 V/mS; SWV frequency: 0.001-100 KHz; DPV and NPV pulse width: 0.0001-1000 s; AD data acquisition: 16 bits-1 MHz, 24bit-100Hz; Minimum potential increment CV: 0.075 mV.

### Microbiological monitoring

Viable counts of SRB, TGB and FB were determined according to the Standard of Petroleum and Natural Gas Industry of People’s Republic of China (Method of SY/T 5890–1993, The national method of the bactericidal agent’s performance). The produced water containing the three kinds of bacteria was gathered from Zichang Oilfield Factory, Yanchang Oilfield.

## Findings

### Inhibitor properties and mechanism

Developing natural products as oilfield chemicals is a straight way to find less expensive, green and eco-friendly materials. Based on the fruitful and large quantity of local plant resources of Shannxi Province, several plants have been investigated in our work for the application in oilfield chemistry. The inhibition efficiency (IE) of extracts of persimmon husk were investigated in the concentration range 10 to 1,000 mg/L in 1 M HCl, and the changes of IE (%) with the inhibitor concentration are summarized in Table 1. From the table, it is apparent that the extracts inhibit the corrosion with different efficiency under different concentrations, the IE increases with increasing extract concentration, and the highest reaches to 65.1%.

**Table 1:** The inhibition efficiency of WE and AE measured by weight loss

**Extract**	**Concentration**	**Corrosion rate**	**Inhibition efficiency**
	**(mg/L)**	**(g/m**^**2**^ **· h)**	**(%)**
__	__	90.6	0
WE	10	60.2	33.6
WE	50	41.9	53.7
WE	100	39.6	56.3
WE	200	38.7	57.2
WE	500	35.3	61.1
WE	1,000	31.6	65.1
AE	10	59.0	34.5
AE	50	52.3	42.0
AE	100	48.1	46.7
AE	200	44.6	50.5
AE	500	39.7	55.9
AE	1,000	38.1	57.7

**Figure 3:**
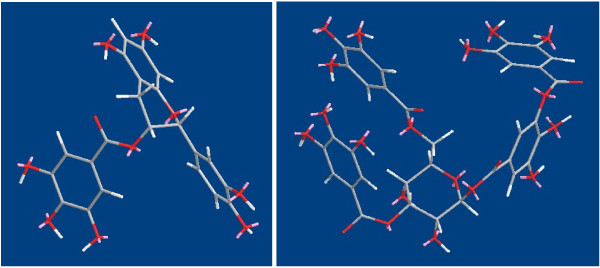
The steady conformation of epicatechin gallate and hydrolysable tannin.

Increasing in IE with increasing additive concentration can be explained on the basis of additive adsorption. The presence of N, O, S atoms and conjugated double bonds in the organic compounds makes the formation of p–d bonds from overlap of p electrons with the vacant 3d orbital of iron atoms, which enhances the adsorption of the compounds on the metal surface [7]. The steady conformations of two phenol compounds in persimmon, epicatechin gallate and hydrolysable tannin, were shown in Figure 3. It is apparent that there are abundant oxygen atoms with couples of p electrons and several conjugated double bonds and aryl groups, which may afford different manner to coordinate with steel surface.

In addition, the phenol compounds are high reductive, and researches have demonstrated that persimmon extract affords protection against lipid peroxidation in *ex vivo* liver homogenates when the tannin of persimmon is added before incubation with Fe^2+^/ascorbic acid or H_2_O_2_[8], which indicate that the phenols can be oxidized to benzoquinone by the O_2_ dissolved in the solution, which can inhibit the oxygen-adsorption corrosion.

### Synergistic effect

KI, KSCN and hexamethylenetetramine (HMTA) are used as synergistic additives in some corrosion inhibitor formulations effectively. In the following work, the effect of KI, KSCN and HMTA on the inhibitive performance of the extracts was studied using weight loss determinations. The IE values under the concentration of 100 mg/L of KI, KSCN and HMTA companied with 500 mg/L of extracts are presented in Table 2. Inspection of Table 3 reveals that KI, KSCN and HMTA they can enhance the IE of WE effectively to as high as 97.3%. But the synergistic effect is not effective for KI, KSCN to AE, only HMTA is effective to give the IE of 90.5%. The reason might lie on the fruitful p-electrons of N, O and triple bond, which can form covalent bonds between the molecules and the ion surface, capture H^+^ to release the acidity and even join the molecules as “bridges” to conform the protective film on the ion surface.

**Table 2:** The corrosion rate inhibition efficiency of extracts companioning with KI, KSCN and HMTA

**Formulation**	**Corrosion rate**	**Inhibition efficiency**
	**(g/m**^**2**^ **· h)**	**(%)**
—	90.6	/
KI	36.1	60.3
KSCN	40.7	55.3
HMTA	24.7	72.8
WE + KI	2.5	97.3
WE + KSCN	8.6	90.6
WE + HMTA	4.9	94.6
AE + KI	21.4	76.5
AE + KSCN	45.1	50.4
AE + HMTA	8.6	90.5

**Table 3:** Potentiodynamic polarization parameters for the corrosion of the Q230A steel in the HCl solution containing WE

**Concentration**	**−Ecorr**	**Icorr**	**βa**	**βc**	**Corrosion rate**	**IE (%)**
**(mg/L)**	**(mV)**	**(μA/cm**^**2**^**)**	**(mV/dec)**	**(mV/dec)**	**(mm/a)**	
--	0.46083	151.440	90.431	155.08	1.7753	--
10	0.46344	89.193	77.592	142.57	1.0491	40.9
50	0.46471	141.990	107.110	166.89	1.5727	11.4
100	0.45503	90.507	77.031	146.09	1.0646	40.0
200	0.45521	31.412	63.159	119.64	0.3695	79.2
500	0.46082	51.929	62.717	81.32	0.6974	60.7
1,000	0.45254	30.770	58.282	135.34	0.36192	79.6

### Tafel polarisation measurements

**Figure 4:**
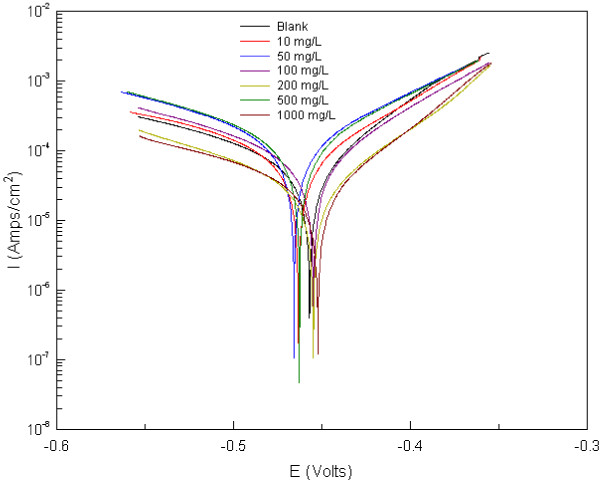
Typical polarization curves for corrosion of Q235A steel in 1 M HCl in the absence and presence of different concentrations of WE.

The anodic and cathodic polarization curves for a mild steel electrode in 1 M HCl in absence and presence of different concentrations of AE at 298 K are shown in Figure 4. Table 3 shows the electrochemical corrosion kinetic parameters, i.e., corrosion potential (*E*_corr_), cathodic and anodic Tafel slopes (*β*a, *β*c) and corrosion current density *I*_corr_ obtained by extrapolation of the Tafel lines. The IE (%) is also calculated from the following equation:

(1) $$E\left(\%\right)=\frac{I_{\mathrm{corr}}-I_{\mathrm{corr}}}{I_{\mathrm{corr}}}\times 100$$

where *I*_corr_ and *I*_corr(i)_ are corrosion current densities obtained in the absence and presence of inhibitors, respectively. As it was expected both anodic and cathodic reactions of mild steel electrode corrosion were inhibited by the increase of the AE. This result suggests that the addition of the WE reduces anodic dissolution and also retards the hydrogen evolution reaction [9]. It can be seen that the corrosion rate is decreased and inhibition efficiency IE is increased by increasing inhibitor concentration. With a concentration of 1,000 mg/L, WE exhibits maximum IE of 79.6%. The extract causes changes in the anodic, cathodic Tafel slopes and the Ecorr values in the presence of different concentrations. *E*_corr_, *β*a and *β*c values do not change appreciably with the addition of the inhibitor, which indicates that the inhibitor is not only interfering with the anodic dissolution or cathodic hydrogen evolution reactions independently but also acts as a mixed-type (anodic/cathodic) of inhibitor [10]. Increasing IE with increasing concentration of the AE shows that the inhibition actions are due to its adsorption on the steel surface [11]. The IEs obtained from potentiodynamic polarization were quite different from those calculated from weight-loss measurements, which is attributable to the fact that the weight-loss method gives average corrosion rate, whereas electrochemical method gives instantaneous corrosion rates. These differences may arise frequently because of the difference in the time required to form an adsorbed layer of inhibitors on metal surface [12].

### Antibacterial activity against oil field microorganism

Produced water is a consequence of oil field exploitation by waterflood or steam injection or having an aquifer linked to the reservoir. The most usual disposal way for the high-volumed produced water is to re-inject it to the well after treatment, which will meet some requirements imposed by environmental and exploitation regulations, among which microbiologically influenced corrosion (MIC) is an example. MIC, mainly caused by the growth of such oil field microorganism as sulfate reducing bacteria (SRB), iron bacteria (IB) and total general bacteria (TGB) in oil pipelines, is considered as a major problem for water treatment in the oil field [13]. Based on this case, different treatment system to inhibit corrosion should be considered, among which using bactericide has received the greatest acceptance. Currently, oxidizer, aldehyde, quatemary ammonium salt and heterocycle compounds, such as Cl_2_, ClO_2_, formaldehyde, pentane-1,5-dial, trichloroisocyanuric acid (TCCA) and ect., have been used as bactericides [14], but the toxicity and oxidation tests have been conducted on a limited selection.

Since tannins have been found to incline to combine with proteins and shown some antibacterial activities [6], the persimmon extracts are anticipated to be bactericides for oil field microorganism. In the following work, the antifungal activity of these extracts against oil field microorganism was tested under the concentrations of 1,000 mg/L and 500 mg/L, and the results are summarized in Table 4. The table showed that extracts are highly antifungal active against the three microorganisms under the concentration of 1000 mg/L. As the concentration of the extracts is reduced to 500 mg/L, the inhibitions are still potent for AE, but the inhibitions of WE are depressed obviously.

**Table 4:** The antifungal activity of persimmon extracts against oil field MIC

**Extract**	**Concentration**	**Microbiotic Concentration/mL**		
	**mg/L**	**iSRB**	**IB**	**TGB**
—	—	110.0	110.0	110.0
WE	500	110.0	110.0	70.0
	1,000	2.0	2.0	1.3
AE	500	2.0	0.6	25.0
	1,000	0.9	0.0	6.0

## Conclusions

The water and alcohol extracts of persimmon husk showed moderate to high effective inhibition in the range 10 to 1,000 mg/L in 1 M HCl at 60°C, and the highest inhibition of 65.1% was obtained by using WE solution of 1,000 mg/L. KI, KSCN and HMTA can enhance the IE of WE effectively to 97.3% at most, but not effective for KI and KSCN to AE. Tafel polarisation measurements indicate the extracts behave as mixed type inhibitor. Investigation of the antibacterial activity against oil field microorganism showed the extracts can inhibit SRB, IB and TGB with moderate to highly efficiency under 1,000 mg/L, which makes extracts potential to be used as bifunctional oil field chemicals.

## Competing interests

The authors declare that they have no competing interests.

## Authors’ contributions

GC has conceived the study, formulated the research idea and prepared the manuscript draft version, JZ and YS carried out the corrosion inhibition experiments, HS and LZ out the Microbiological monitoring, and JZ participated in its design and coordination. All authors have read and approved the final manuscript.
